# Comprehensive evaluation of individual and combined dietary carotenoids in *Penaeus vannamei* and response surface analysis for optimizing combinations

**DOI:** 10.3389/fimmu.2025.1688761

**Published:** 2025-10-15

**Authors:** Yucai Xue, Bin Han, Yansong Xue, Ganfeng Yi, Meiqin Wu, Amina S Moss, Xuxiong Huang, Weilong Wang

**Affiliations:** ^1^ Building of China-ASEAN Belt and Road Joint Laboratory on Mariculture Technology, Shanghai, China; ^2^ Centre for Research on Environmental Ecology and Fish Nutrition of the Ministry of Agriculture, Shanghai, China; ^3^ National Demonstration Center for Experimental Fisheries Science Education, Shanghai Ocean University, Shanghai, China; ^4^ Institute of Aquaculture, University of Stirling, Scotland, United Kingdom; ^5^ Key Laboratory of Aquaculture Nutrition and Feed (Ministry of Agriculture and Rural Affair), Key Laboratory of Mariculture (Ministry of Education), Ocean University of China, Qingdao, China

**Keywords:** carotenoids, Penaeus vannamei, optimizing combinations, AHP-CRITIC-TOPSIS, response surface analysis

## Abstract

Carotenoids are essential in crustacean aquaculture, supporting pigmentation, antioxidant defense, immune function, and sensory quality. However, intensive culture restricts natural carotenoid inputs, and exclusive reliance on astaxanthin is constrained by cost and supply. Evidence to guide cost-effective formulation remains fragmented: reported optimal doses vary widely, head-to-head comparisons under a common basal diet are scarce, mixture effects are poorly defined, and rigorous multi-factor optimization is rare. In this study, *Penaeus vannamei* were fed iso-nitrogenous, iso-lipidic diets for 56 days that supplemented β-carotene, canthaxanthin, or astaxanthin individually, or all three in combination at literature-based levels. Outcomes included growth and feed efficiency; tissue carotenoid deposition and composition; antioxidant and digestive enzyme activities; color (CIELAB); and flavor (free amino acids, nucleotides, organic acids). A subsequent response-surface experiment varied inclusion ranges (β-carotene 200-500, canthaxanthin 100-300, astaxanthin 50–250 mg/kg) to model total astaxanthin, total carotenoids, total antioxidant capacity, and redness, followed by AHP-CRITIC-TOPSIS multi-criteria decision analysis to identify the global optimum.All carotenoid diets increased final weight and specific growth rate and reduced feed conversion ratio relative to the control, with the mixture outperforming any single carotenoid. Supplementation elevated digestive enzyme activities and enhanced antioxidant status (higher T-AOC, lower malondialdehyde), with astaxanthin the most efficient single additive and the mixture providing additional gains. Astaxanthin (> 80% of tissue carotenoids) accumulated mainly in the hepatopancreas; β-carotene promoted greater total carotenoid and astaxanthin deposition than direct astaxanthin, and the mixture produced the highest tissue levels. Carotenoids improved color (lower L*, higher a*) with a plateau in redness, and increased umami-related free amino acids, nucleotides, and succinic acid, thereby raising the equivalent umami concentration (EUC). Optimization identified a β-carotene: canthaxanthin: astaxanthin ratio of 368: 204.5: 219.2 mg/kg as the best composite solution.This work provides a rigorously benchmarked and optimization-based formulation strategy, yielding actionable inclusion ratios that reconcile biological performance with cost-effective feed design for intensive shrimp culture.

## 1 Introduction

Carotenoids are lipid-soluble pigments with multifunctional roles in crustaceans, shaping body coloration and market value while supporting antioxidant capacity, immune competence, and tolerance to environmental stress (1). Farmed penaeids, including the Pacific white shrimp *Penaeus vannamei*, cannot synthesize carotenoids *de novo* and therefore rely on dietary sources to meet physiological demands (2, 3). Under intensive culture, simplified trophic inputs and variable natural pigment availability often cause carotenoid insufficiency, impairing performance and product quality (4, 5). These constraints have motivated targeted carotenoid supplementation in shrimp feeds.

Among available options, astaxanthin is widely regarded as the benchmark because of its strong pigmentation capacity and documented benefits for growth and health (2). However, its cost and supply volatility motivate evaluation of complementary or alternative carotenoids that achieve comparable outcomes at lower cost (6). β-carotene and canthaxanthin are two such candidates with established use in aquafeeds (7). Beyond their individual bioactivity, these carotenoids are metabolically connected: β-carotene can be enzymatically converted to more oxygenated xanthophylls-including canthaxanthin and astaxanthin-and canthaxanthin may act as an intermediate in astaxanthin formation within crustacean tissues (8). This connectivity implies that mixtures could reshape tissue pigment pools and physiological responses in ways that single-compound studies may not capture (4).

Carotenoids play diverse physiological roles in penaeid shrimp, including growth promotion, pigmentation, antioxidant defense, and immune modulation. Reported optimal supplementation levels for individual carotenoids have been documented, with astaxanthin at 100–200 mg/kg (9), β-carotene at 300–400 mg/kg (6), and canthaxanthin at 173.73–202.13 mg/kg (8). Despite extensive research on single-carotenoid supplementation in penaeids, several practical questions remain unresolved. Reported “optimal” inclusion levels vary widely, influenced by shrimp size, basal diet, matrix effects, and the choice of endpoint (e.g., growth, pigmentation, or health) (2, 10). Direct head-to-head comparisons under a unified dietary background are scarce, complicating efforts to rank carotenoids on a common performance scale. Moreover, mixtures are seldom evaluated for additivity or synergy, despite their plausibility in commercial formulations (7). Critically, formal multifactor optimization that accommodates nonlinearity and interactions is rare.

To address these gaps, we conducted a two-stage investigation in *P. vannamei* using semi-purified, iso-nitrogenous and iso-lipidic diets to isolate carotenoid effects. First, the current study benchmarked β-carotene, canthaxanthin, and astaxanthin-alone and in combination-on growth performance, pigment deposition/coloration, and health-related indicators under controlled conditions. Second, the current study applied response-surface methodology (RSM) to map main and interactive effects across graded inclusion levels and to predict optimal combinations. By integrating direct comparisons with a formal optimization framework, the current study aims to identify cost-conscious carotenoid strategies that sustain growth and product quality in intensive culture and to provide actionable guidance for evidence-based formulation of *P. vannamei* feeds.

## 2 Materials and methods

### 2.1 Carotenoid sources and experimental diet preparation

Experimental 1: Five iso-nitrogenous (~42% crude protein) and iso-lipidic (~8% crude lipid) semi-purified pelleted diets were formulated (**Table 1**). The control diet contained no carotenoid supplementation, while the four experimental diets were supplemented with: β-carotene (350 mg/kg), astaxanthin (150 mg/kg), canthaxanthin (200 mg/kg), and a combination of β-carotene (350 mg/kg), astaxanthin (150 mg/kg), and canthaxanthin (200 mg/kg) (**Table 2**). Carotenoid inclusion levels were based on optimal values reported in previous studies (6, 8). The carotenoid sources were: (1) β-carotene (β-car): Synthetic β-carotene (POVIMIX^®^ β-Carotene 10%; DSM Nutrition, Heerlen, Switzerland), containing 10% (w/w) β-carotene; (2) Astaxanthin (Ast): Synthetic astaxanthin (CAROPHYLL^®^ Pink; DSM Nutrition, Heerlen, Switzerland), containing 10% (w/w) astaxanthin; (3) Canthaxanthin (Can): Synthetic canthaxanthin (CAROPHYLL^®^ Red; DSM Nutrition, Heerlen, Switzerland), containing 10% (w/w) canthaxanthin. Casein and crystalline amino acid were included to meet the protein and amino acid balance requirements, while fish oil, soybean lecithin, and cholesterol served as primary lipid sources. Additional ingredients were incorporated to satisfy other essential dietary requirements.

**Table 1:** Formulation and biochemical composition of the experimental diets (g/kg, dry matter basis).

Parameters	Treatments				
	Control	β-car	Ast	Can	Mix
Casein^a^	385	385	385	385	385
L-lysine^b^	15	15	15	15	15
DL-methionine^b^	10	10	10	10	10
L-arginine^b^	20	20	20	20	20
Fish oil^a^	48	48	48	48	48
Soybean lecithin^a^	30	30	30	30	30
Cholesterol^b^	5	5	5	5	5
Vitamin mixture^c^	6.5	6.5	6.5	6.5	6.5
Mineral mixture^d^	34	34	34	34	34
a-Starch^a^	30	30	30	30	30
Sucrose^b^	50	50	50	50	50
Glucose^b^	50	50	50	50	50
Glucosamine-HCl^b^	8	8	8	8	8
Sodium citrate^b^	3	3	3	3	3
Sodium succinate^b^	3	3	3	3	3
Wheat flour^a^	235	235	235	235	235
Ca(H_2_PO_4_)_2_ ^a^	52	52	52	52	52
Cellulose^a^	10	6.5	8.5	8	3
Choline choride^a^	5.5	5.5	5.5	5.5	5.5
β-carotene^e^	0	3.5	0	0	3.5
Astaxanthin^f^	0	0	1.5	0	1.5
Canthaxanthin^g^	0	0	0	2	2
Total	1000	1000	1000	1000	1000

a Yuehai Feed Group Co., Ltd., Zhejiang, China.

b Shanghai Macklin Biochemical Co., Ltd., Shanghai, China.

c Vitamin mixture (6.5 g/kg diet) (vitamin A free): p-aminobenzoic acid 0.092 g; biotin 0.004 g; inositol 3.668 g; nicotinic acid 0.368 g, Ca-pantothenate 0.552 g; pyridoxine-HCl 0.112 g; riboflavin 0.072 g; thiamine-HCl 0.036 g; menadione 0.036 g, α-tocopherol 0.184 g; cyanocobalamin 0.0008 g; cholecalciferol 0.012 g; stay-C 1.36 g, folic acid 0.008 g.

d Mineral mixture (34 g/kg diet): C_6_H_10_CaO_6_·5H_2_O, 1.73 g; K_2_HPO_4_, 11.84 g; MgSO_4_.7H_2_O, 12.14 g; NaH_2_PO_4_, 6.96 g; C_6_H_5_O_7_Fe·5H_2_O, 0.23 g; CuSO_4_.5H_2_O, 0.34 g; ZnSO_4_·7H_2_O, 0.48 g; CoCl_2_, 0.07 g; MnSO_4_·H_2_O, 0.21 g.

e β-carotene (350 mg/kg): POVIMIX® β-Carotene containing 10 % Synthetic β-carotene made by DSM Nutrition, Heerlen, Switzerland.

f Astaxanthin (150 mg/kg): CAROPHYLL® Pink containing 10 % Synthetic astaxanthin made by DSM Nutrition, Heerlen, Switzerland.

g Canthaxanthin (250 mg/kg): CAROPHYLL® Red containing 10 % Synthetic canthaxanthin made by DSM Nutrition, Heerlen, Switzerland.

**Table 2:** Biochemical composition analysis of experimental diets (dry mater basis).

Parameters^1^	Treatments				
	Control	β-car	Ast	Can	Mix
Crude protein (%)	41.58 ± 0.35	41.32 ± 0.44	41.26 ± 0.13	41.44 ± 0.22	41.28 ± 0.31
Crude lipid (%)	8.19 ± 0.16	8.33 ± 0.36	8.05 ± 0.25	8.42 ± 0.57	8.29 ± 0.48
Ash (%)	9.06 ± 0.28	8.84 ± 0.21	8.96 ± 0.15	9.01 ± 0.31	8.79 ± 0.16
Moisture (%)	9.85 ± 0.25	10.01 ± 0.45	9.64 ± 0.41	9.34 ± 0.33	10.14 ± 0.15
β-carotene (mg/kg)	ND	345.19 ± 2.32	ND	ND	348.25 ± 1.79
Astaxanthin (mg/kg)	ND	ND	144.14 ± 1.22	ND	147.23 ± 0.81
Canthaxanthin (mg/kg)	ND	ND	ND	196.42 ± 1.73	198.33 ± 2.17

^1^Values are expressed as means ± S.E.M. (n = 3). Different superscript letters within the same row indicate statistically significant different (*P* < 0.05). ND, not detected.

Experimental 2: Eighteen iso-nitrogenous (~42% crude protein) and iso-lipidic (~8% crude lipid) semi-purified pelleted diets were formulated using identical ingredient sources and basal diet compositions. The control diet contained no carotenoids, while experimental diets included varying levels of β-carotene (200, 350, and 500 mg/kg), astaxanthin (50, 150, and 250 mg/kg), and canthaxanthin (100, 200, and 300 mg/kg) A response surface methodology was employed using Design-Expert version 13 (Stat-Ease Inc., Minneapolis, USA) to determine the optimal carotenoid combination (**Supplementary Table S1**).

Diet preparation: All dry ingredients were finely ground (< 150 μm) and thoroughly mixed. Premixed lipid components and fat-soluble ingredients were then incorporated. Water (25% of the dry mix wight) was gradually added to form a homogeneous dough, which was extruded through a single-screwed mincer to produce 1.5 mm diameter pellets. Pellets were initially dried in a mechanical convection oven at 90 °C for 20 minutes to facilitate starch gelatinization, followed by drying at 35 °C until the moisture content reached 10%. Diets were sealed in plastic bags and stored at -20 °C until use.

### 2.2 Feeding trial and experimental conditions

Shrimp at post-larval stage 7 (P7) were obtained from a local hatchery (Haixingnong Aquaculture Cooperative, Shanghai, China), where the salinity had been gradually reduced from 20 ‰ to 2‰ during the post-larval stage. The shrimp were subsequently reared in nursery ponds, acclimated to the culture conditions for three weeks, and fed the control diet prior to the start of feeding trial.

For experiment 1, a total of 600 juveniles (average initial weight: 0.25 ± 0.02 g) were randomly allocated to 15 indoor 500 L PVC tanks (height = 117 cm, radius = 50 cm) connected to a recirculating system equipped with a biological filter. Each treatment was replicated three times, with forty shrimp per tank. For experiment 2, 2160 juveniles were distributed among 54 indoor tanks of the same specifications.

The feeding trial lasted eight weeks. Shrimp were hand-fed four times daily (7:00, 12:00, 17:00, and 22:00) at a total rate of 5-8% of body weight (8). Feeding rates were adjusted weekly based on body weight gain determining through random sampling. Uneaten feed and fecal matter were regularly removed, and all unconsumed feed collected, freeze-dried, and used to calculate feed intake and feed efficiency ratios.

Water quality was monitored daily to maintain optimal conditions: water temperature was kept at 28-31 °C, dissolved oxygen above 6 mg/L, ammonia nitrogen below 0.05 mg/L, pH between 7.8 to 8.5, and salinity at approximately 2-3 ‰. Concentration of NH_4_^+^-N, NO_3_^-^-N, and NO_2_^-^-N were determined using Nessler’s reagent colorimetry, phenol disulfonic acid method, and N-(1-naphthalene)-diaminoethane spectrophotometry, respectively, following Yan et al. (11).

### 2.3 Samples collection and growth performance calculation

At the end of the 8-week feeding trial, juveniles were fasted for 12 hours prior to final sampling, following the protocol of Fawzy et al. (6). The total number of surviving juveniles and their body weights were recorded. From each experimental group, fifteen shrimp (five per replicate) were randomly selected for whole-body composition and carotenoid content analyses. Hemolymph samples were collected from the ventral sinus of nine shrimp per replicate using a 1-mL syringe. The hepatopancreas and intestine were dissected from each individual and placed into 2-mL tubes. All hemolymph, intestine, and hepatopancreas samples were immediately flash-frozen in liquid nitrogen and subsequently stored at -80 °C until further analysis. Additionally, five shrimp from each replicate were used for pigmentation assessment.

Growth performance indicators-including weight gain (WG), specific growth rate (SGR), condition factor (CF), survival rate, and feed conversion ratios (FCR)-were calculated according to the formulas described by Xue et al. (1):


WG (%) = [(final weight−initial weight)/initial weight] × 100;

$$\begin{array}{c} \text{SGR }\left(\%/\text{day}\right)\;=\;\left[\left(\text{Ln final weight}-\text{Ln initial weight}\right)/\text{duration}\right]\;\\ \times \;100; \end{array}$$

$$\begin{array}{c} \text{Survival }\left(\%\right)\;\\ =\;\left(\text{final number of shrimp}/\text{initial number of shrimp}\right)\;\times \;100; \end{array}$$

FCR = dry weight of feed consumed (g)/live weight gain (g).

### 2.4 Proximate composition analysis

Whole-body and diet samples were freeze-dried, ground, and analyzed for proximate composition following to the methods of the Association of Official Analytical Chemists (12). Crude protein (N factor = 6.25) was determined using the Dumas method (AOAC method 990.03) with a Dumas Nitrogen Analyzer (Model CHN828, LECO Corporation, Michigan, USA). Crude lipid was extracted following the method of Folch et al. (13) and quantified gravimetrically after drying a 5 mL aliquot under nitrogen. Ash content was measured by pre-incineration on a hot plate, followed by combustion in a muffle furnace (Model KSL-1200X, Hefei, China) at 550 °C for six hours (AOAC 942.05). Moisture content was determined by oven drying (Model DHG-9245A, Bluepard Instruments, Shanghai, China) at 105 °C for 5 hours (AOAC 935.29).

### 2.5 Activities of hepatopancreatic antioxidant parameters and digestive enzymes

Hepatopancreas samples were mixed with shrimp saline solution at a 1:5 ratio, homogenized, and centrifuged at 8,000 rpm for 15 minutes at 4 °C using a refrigerated centrifuge (Model TG-16, Cence, Hunan, China). The resulting supernatants were used for antioxidant and digestive enzyme assays following the methods of Xue et al. (14).

Antioxidant parameters: Total antioxidant capacity (T-AOC), peroxidase (POD), glutathione peroxidase (GSH-Px), malondialdehyde (MDA), and catalase (CAT) were measured using commercial kits (Nanjing Jiancheng Bioengineering Institute, Nanjing, China) according to the manufacturer’s instructions. T-AOC was determined by the ABTS method (Kit A084-1-1), in which ABTS reacts with an oxidizing agent to generate ABTS^+^, and antioxidants inhibits this reaction. Absorbance was recorded to quantify total antioxidant capacity. POD activity (Kit A084-3-1) was determined colorimetrically at 420 nm, based on the oxidation of a substrate catalyzed by POD in the presence of hydrogen peroxide. GSH-Px activity (Kit A005-1-2) was determined by measuring the rection between hydrogen peroxide (H_2_O_2_) and reduced glutathione (GSH), with GSH consumption quantified via its reaction with Ellman’s reagent to form a yellow product. MDA content (Kit A003-1-2) was quantified using the thiobarbituric acid (TBA) method, in which MDA reacts with TBA to form a red product measured at 532 nm. CAT activity (Kit A007-1-1) was assessed colorimetrically at 405 nm by measuring the decomposition of H_2_O_2_ by catalase, stopped with ammonium molybdate to form a yellow complex.

Digestive enzymes: Protease, lipase, and amylase activities were also analyzed using commercial kits (Nanjing Jiancheng Bioengineering Institute, Nanjing, China). Protease activity (Kit A080-1-1) was determined colorimetrically by measuring the absorbance of blue complexes formed between amino acids (produced during protein hydrolysis) and Folin-phenol reagent. Lipase activity (Kit A054-1-1) was measured based on the turbidity change resulting from triglyceride hydrolysis, which disrupts micelle structure and reduces light scattering. Amylase activity (Kit C016-1-1) was determined by hydrolyzing starch into glucose, maltose, and dextrin with α-amylase. Iodine was then added to react with residual starch, forming a blue complex; the reduction in color intensity corresponded to the extent of starch hydrolysis and enzyme activity.

### 2.6 Carotenoid contents and pigmentation determination

Carotenoid contents in shrimp shells (including carapace, telson, and uropod), muscle, whole shrimp, and experimental diets were quantified using a Waters ACQUITY UPLC System (Waters Corporation, Milford, USA) equipped with a Waters ACQUITY H-Class BEH C_18_ column (1.7 μm, 2.1 mm × 150 mm, Waters Corporation, Milford, MA, USA), following the method of Fawzy et al. (6). Briefly, carotenoids were extracted from lyophilized tissue powder using an acetone–methanol solution (v/v, 2:1). The crude extract was dried under nitrogen, re-dissolved in the mobile phase, and directly analyzed by UPLC to determine free astaxanthin and other carotenoids. For total carotenoid determination, the crude extract was saponified with methanolic NaOH (final concentration 0.02 M) for 12 h, followed by extraction with an equal volume of deionized water and chloroform. The chloroform layer was collected, dried under nitrogen, re-dissolved in the mobile phase, and analyzed under the same chromatographic conditions. The difference between total and free astaxanthin was considered the esterified fraction.

Shrimp pigmentation was evaluated using a handheld colorimeter (Chroma Meter CR-400, Konica Minolta Sensing Inc., Osaka, Japan). Each shrimp was boiled for 3 minutes in a boiling water, immediately cooled in tap water (~5 °C) in the dark, and then subjected to color measurement. Measurements were performed under standardized fluorescent as described by Wang et al. (15). The color parameters-*L* ^*^ (lightness), *a* ^*^ (redness), and *b* ^*^ (yellowness)-were recorded according to the International Commission on Illumination (CIE) color space standards (16).

### 2.7 Determination of non-volatile flavor substances

Free amino acid content: Free amino acid content in shrimp muscle was determined according to Zhang et al. (17) with slight modifications. Briefly, 0.5 g of each sample was homogenized with 15 mL of 5% trichloroacetic acid (TCA) for 120 seconds, sonicated for 30 minutes, and left to stand for 2 hours. The mixture was centrifuged at 10,000 rpm for 15 minutes at 4 °C. A 5 mL aliquot of the supernatant was collected, the pH adjusted to 2.0 using 1 mol/L sodium hydroxide, diluted to 10 mL with 5% TCA, filtered through a 0.22 μm aqueous membrane, and analyzed using an automatic amino acid analyzer (LA8080, Hitachi, Tokyo, Japan).

Nucleotide content: Nucleotide content was determined following Zhang et al. (17) with minor modifications. A 3 g muscle sample was homogenized with 10 mL of 10% cold perchloric acid for 60 seconds, sonicated for 5 minutes at low temperature, and centrifuged at 10,000 rpm for 15 minutes at 4 °C. The residue was re-extracted twice with 5 mL of 5% perchloric acid under the same conditions. Combined supernatants were adjusted to pH 5.75 ± 0.02 using 6 mol/L or 1 mol/L potassium hydroxide, refrigerated at 4 °C for 30 minutes, diluted to 50 mL, and filtered through a 0.22 μm aqueous membrane. Quantification was using a Waters e2695 HPLC System (Waters Corporation, Milford, USA) equipped with a reverse-phase Inertsil ODS-E C18 column (5 μm, 4.6 mm × 250 mm, GL Sciences, Tokyo, Japan).

Organic acid content: Organic acids was analyzed according to Duan et al. (18) with slight modifications. 1 g of muscle tissue was homogenized with 20 mL of ultrapure water at 4 °C (9,000 rpm, 10 min), centrifuged, and filtered through a 0.22 μm membrane. Analyses were performed on a Waters e2695 HPLC system (Waters Corporation, Milford, USA) equipped with a Cosmosil MS-II C_18_ column (5 μm, 4.6 mm × 250 mm, NACALAI TESQUE, Kyoto, Japan) maintained at 30 °C. Isocratic separation employed 2% diammonium phosphate buffer (pH 2.9) at a flow rate of 0.8 mL/min, and detection was carried out at 214 nm.

The taste activity value (TAV) was calculated as described in the equation. Compounds with a TAV greater than 1 are generally considered to contribute to the overall taste profile (19).


$$TAV=\frac{C}{T}$$

In the equation, C represents the absolute concentration of the flavored compound (mg/100 g), and T denotes its sensory threshold (mg/100 g).

The equivalent umami concentration (EUC) quantifies the synergistic effect of umami amino acids and 5′- flavor nucleotides on taste perception, and was calculated using the following equation:


EUC=∑aibi+1218(∑aibi)(∑ajbj)

EUC is expressed as grams of monosodium glutamate (MSG) per 100 g of sample. In the equation: ai represents the concentration of umami amino acids (g/100 g), with bi as their corresponding relative umami coefficients (Glu, 1; Asp, 0.077); aj denotes the concentration of 5′-nucleotides (g/100 g), and bj their respective umami coefficients (IMP, 1; GMP, 2.3; AMP, 0.18); 1218 is the synergistic constant used to account for the interaction between amino acids and nucleotides.

### 2.8 Statistical analysis and visualization

The data were tested for normality and homogeneity of variances using the Shapiro-Wilk test and Levene’s test, respectively. For datasets meeting these assumptions, a one-way ANOVA was performed using SPSS 20.0 to evaluate the effects of dietary carotenoid supplementation on *P. vannamei*, followed by Duncan’s multiple range test for *post hoc* comparisons. Data that violated the assumptions were log-transformed to satisfy parametric tests requirements. Differences among treatment means were considered significant at *P* < 0.05. Results are presented as means ± standard error of the mean (S.E.M., n = 3). Visualization was generated using the ggplot2 package (version 3.5.1) in R (version 4.4.2). Final figures were prepared in Adobe illustrator (version 29.1).

### 2.9 Optimization of Formulation by AHP-CRITIC-TOPSIS

In this study, RSM was employed to develop predictive function g(x) for each objective response as a function of independent variables: β-carotene ( $x_{1}\in [200,500]$ ), canthaxanthin ( $x_{2}\in [100,300]$ ), and astaxanthin ( $x_{3}\in [50,250]$ ). Objective weights for the indicators were calculated using a modified CRITIC algorithm, where the standard deviation was replaced with the coefficient of variation ( $CV_{j}=\sigma_{j}/\mu_{j}$ ) to better capture relative dispersion:


$$C_{j}=CV_{j}\sum_{k=1}^{J}(1-r_{jk}),\;\;w_{j}^{CRITIC}=\frac{C_{j}}{\sum_{j=1}^{J}C_{j}}$$

Here, $r_{jk}$ denotes the Pearson correlation coefficient between indicators j and k .

This coefficient quantifies the degree of conflict or redundancy among indicators: stronger correlations imply lower conflict and reduced uniqueness in the conveyed information (20).

Subjective weights ( $W_{S}$ ​) were derived from expert comparison matrices using Saaty’s Analytic Hierarchy Process (AHP) (21). Consistency ratios (CR) were required to be below 0.10. The final composite weight for indicators h was determined by the geometric mean of subjective and objective weights:


$$W_{C}^{\left(h\right)}\;=\frac{\sqrt{W_{S}^{\left(h\right)}W_{O}^{\left(h\right)}}}{\sum_{h}\sqrt{W_{S}^{\left(h\right)}W_{O}^{\left(h\right)}}}$$

A comprehensive multi-indicator evaluation function ( f(x) ) was developed by integrating the response surface prediction model ( g(x) ) with the Technique for Order Preference by Similarity to Ideal Solution (TOPSIS), thereby identifying the optimal comprehensive composite solution:


$$f(x)=\frac{\sqrt{\sum_{j=1}^{m}w_{i}{\left(\frac{g_{i}(x)-A_{i}^{+}}{A_{i}^{+}-A_{i}^{-}}\right)}^{2}}}{\sqrt{\sum_{j=1}^{m}w_{i}{\left(\frac{g_{i}(x)-A_{i}^{+}}{A_{i}^{+}-A_{i}^{-}}\right)}^{2}}+\sqrt{\sum_{j=1}^{m}w_{i}{\left(\frac{g_{i}(x)-A_{i}^{+}}{A_{i}^{+}-A_{i}^{-}}\right)}^{2}}}$$

$A_{i}^{+}$ : Positive ideal value of the i -th indicator, $A_{i}^{-}$ : Negative ideal value of the i -th indicator.

## 3 Results

### 3.1 Growth performance, survival, and feed utilization

Growth outcomes for *P. vannamei* over the 56-day trial are summarized in **Table 3**. All carotenoid-supplemented diets produced significantly higher final body weight (FBW), weight gain (WG), and specific growth rate (SGR), and a significantly lower feed conversion ratio (FCR), than the control (*P* < 0.05). Among the supplemented treatments, the carotenoid mixture yielded significantly greater growth performance than any single-carotenoid diet (*P* < 0.05). Survival rate (SR) did not differ among treatments (*P* > 0.05).

**Table 3:** Growth parameters and survival rate of *P. vannamei* fed with experimental diets for 56 days.

Parameters^1^	Treatments					*P*-value
	Control	β-car	Ast	Can	Mix	
FBW (g)	10.12^c^ ± 0.26	11.14^b^ ± 0.32	11.87^b^ ± 0.51	11.01^b^ ± 0.39	12.45^a^ ± 0.43	0.015
WG (%)	3914.02^c^ ± 39.57	4238.21^b^ ± 83.48	4255.82^b^ ± 72.26	4105.93^b^ ± 39.67	4430.69^a^ ± 55.36	0.025
SGR (%)	7.80^c^ ± 0.13	8.38^b^ ± 0.07	8.45^b^ ± 0.08	8.17^b^ ± 0.14	8.95^a^ ± 0.03	0.032
Survival (%)	88.63 ± 3.75	86.67 ± 3.63	88.00 ± 2.31	87.50 ± 1.44	85.83 ± 2.5	0.243
FCR	1.64^a^ ± 0.05	1.49^b^ ± 0.02	1.42^b^ ± 0.05	1.51^b^ ± 0.03	1.33^c^ ± 0.03	0.033

^1^ Values are expressed as means ± S.E.M. (n = 3). Different superscript letters within the same row indicate statistically significant different (*P* < 0.05). FBW, final body weight; WG, weight gain; SGR, specific growth rate; FCR, feed conversion ratio.

### 3.2 Biochemical analysis composition of shrimp whole-body

The whole-body proximate composition of *P. vannamei* fed different experimental diets over a 56-day period is summarized in **Table 4**. No significant differences were detected among treatments in crude protein, ash, or moisture contents (*P* > 0.05). However, total lipid content was significantly higher in all carotenoid-supplemented groups compared to the control (*P* < 0.05), with the carotenoid mixture group exhibiting the significant highest whole-body crude lipid level.

**Table 4:** Whole-body proximate analysis (%, wet weight) of *P. vannamei* fed with experimental diets for 56 days.

Parameters^1^	Treatments					*P*-value
	Control	β-car	Ast	Can	Mix	
Crude protein (%)	17.58 ± 0.37	17.32 ± 0.42	17.26 ± 0.23	17.44 ± 0.26	17.28 ± 0.35	0.653
Crude lipid (%)	1.19^c^ ± 0.06	1.46^b^ ± 0.06	1.51^b^ ± 0.17	1.43^b^ ± 0.17	1.73^a^ ± 0.08	0.021
Ash (%)	2.53 ± 0.18	2.84 ± 0.11	2.46 ± 0.19	2.33 ± 0.31	2.79 ± 0.26	0.342
Moisture (%)	73.83 ± 0.45	74.01 ± 0.65	74.64 ± 0.41	75.34 ± 0.83	74.14 ± 1.10	0.244

^1^ Values are expressed as means ± S.E.M. (n = 3). Different superscript letters within the same row indicate statistically significant different (*P* < 0.05).

### 3.3 Digestive enzyme activities

**Figure 1:**
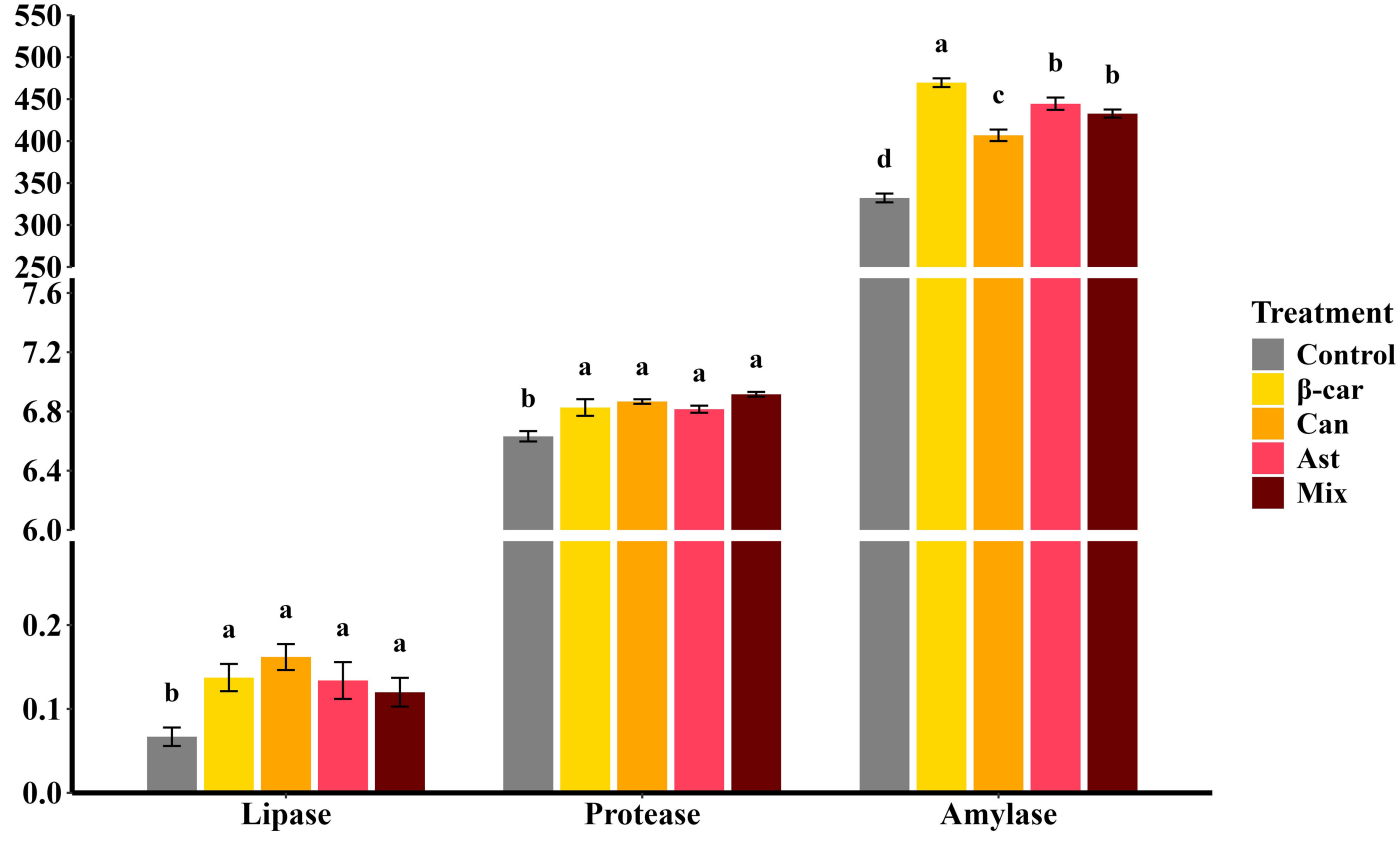
Effect of dietary different carotenoids on hepatopancreatic digestive enzyme activities (U/mg protein) in *P. vannamei.* Values are expressed as means ± S.E.M. (n = 3). Bars with different letters represent significant differences among various treatments (*P* < 0.05). ANOVA *P* > F: Lipase (0.025), Protease (0.001), Amylase (< 0.001).

After the 56-day feeding trial, hepatopancreatic digestive-enzyme activities of *P. vannamei* (**Figure 1**) were significantly elevated by dietary β-carotene, astaxanthin, and canthaxanthin compared with the control (*P* < 0.05). Among the single-supplement diets, lipase and protease activities did not differ (*P* > 0.05), whereas amylase activity was highest with β-carotene, followed by astaxanthin. Combined carotenoid supplementation further improved overall digestive-enzyme activities (*P* < 0.05); however, lipase and protease in the combined group were not different from the single-supplement groups, and amylase did not differ from the astaxanthin group (*P* > 0.05).

### 3.4 Hepatopancreatic antioxidant enzyme activities

**Figure 2:**
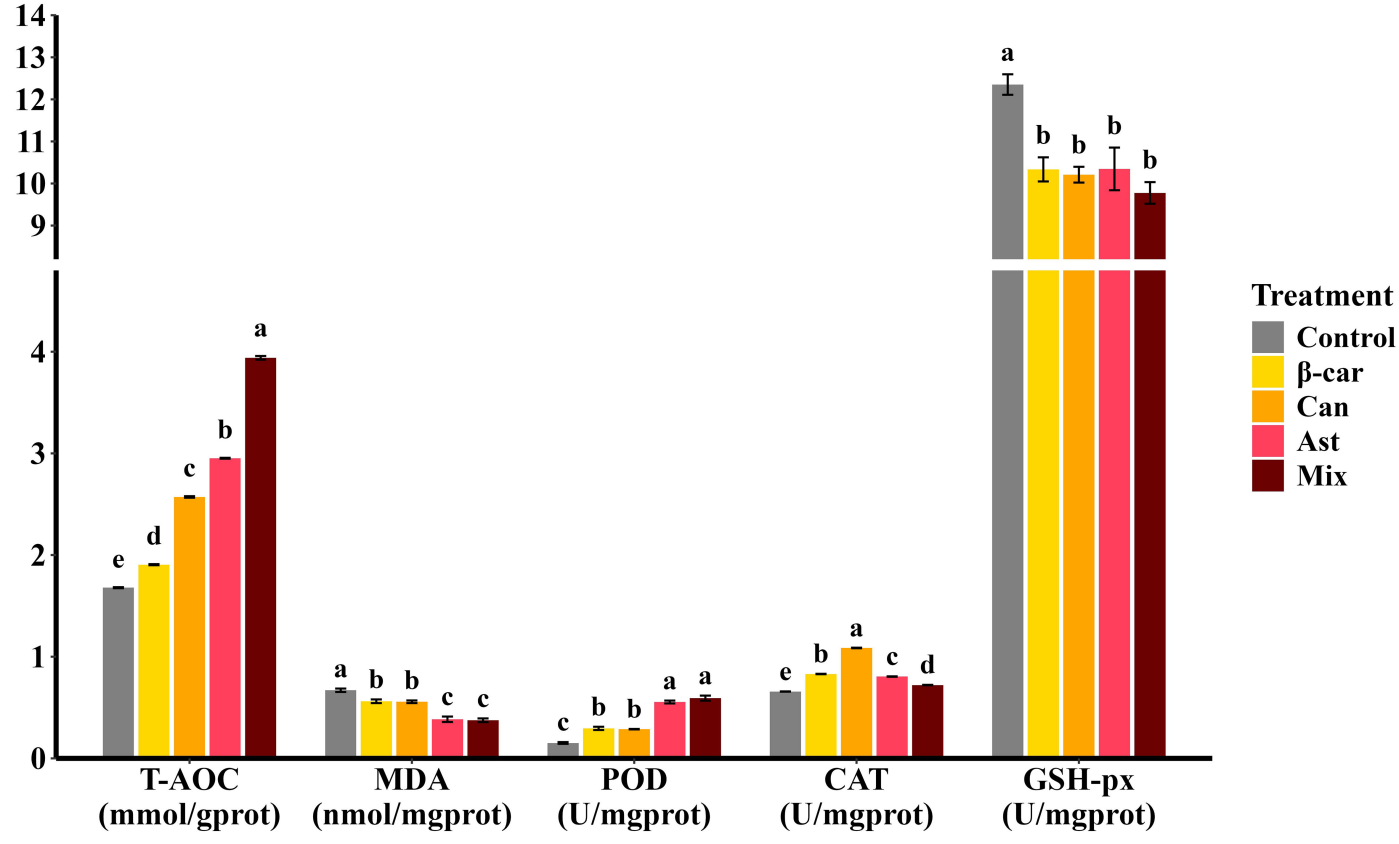
Effect of different dietary carotenoids on hepatopancreatic antioxidant status in *P. vannamei.* Values are expressed as means ± S.E.M. (n = 3). Bars with different letters represent significant differences among various treatments (*P* < 0.05). T-AOC, total antioxidant capacity; MDA, malondialdehyde; CAT, catalase; GSH-Px, glutathione peroxidase; POD, peroxidase. ANOVA *P* > F: T-AOC (< 0.001), MDA (< 0.001), CAT (< 0.001), GSH-Px (< 0.001), POD (0.001).

After the 56-day feeding trial, hepatopancreatic antioxidant metrics of *Penaeus vannamei* (**Figure 2**) responded strongly to dietary carotenoids. Supplementation with β-carotene, astaxanthin, or canthaxanthin increased total antioxidant capacity (T-AOC) and reduced malondialdehyde (MDA) relative to the control (*P* < 0.05). Among single-supplement diets, astaxanthin produced the highest T-AOC and the lowest MDA. The combination diet further elevated T-AOC above astaxanthin alone (*P* < 0.05), whereas MDA did not differ from the astaxanthin treatment (*P* > 0.05). Enzyme-specific assays showed higher peroxidase (POD) and catalase (CAT) activities and lower glutathione peroxidase (GSH-Px) activity with carotenoid supplementation; the combined diet followed the same pattern.

### 3.5 Carotenoids composition of shrimp

**Figure 3:**
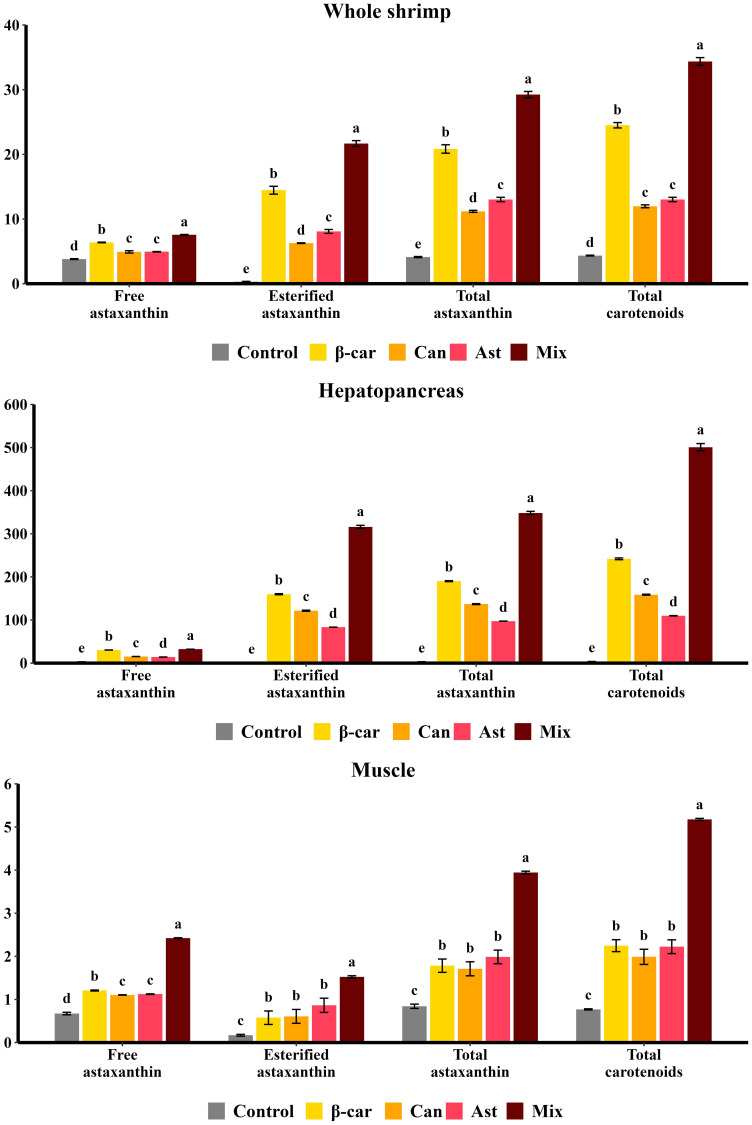
Effect of different dietary carotenoids on carotenoid compositions (mg/kg) in *P. vannamei.* Values are expressed as means ± S.E.M. (n = 3). Bars with different letters represent significant differences among various treatments (*P* < 0.05). Exact ANOVA *P* values are reported in **Supplementary Table S2**.

Astaxanthin and total carotenoid concentrations and composition in whole shrimp, hepatopancreas, and muscle after 56 days are shown in **Figure 3**. Astaxanthin was the predominant pigment, accounting for > 80% of total carotenoids. In controls (no dietary carotenoids), astaxanthin occurred mainly in the free form; with supplementation, it was predominantly esterified, especially in the hepatopancreas. The hepatopancreas was the principal deposition site, with carotenoid levels ~10-fold higher than in whole shrimp and ~100-fold higher than in muscle. Among single-carotenoid diets at their optimal inclusion levels, β-carotene produced significantly higher totals of astaxanthin and carotenoids in whole shrimp and hepatopancreas than canthaxanthin or astaxanthin (*P* < 0.05). Muscle totals did not differ among treatments (*P* > 0.05). The combination diet further increased carotenoid accumulation beyond single-supplement maxima, yielding significantly higher concentrations in all tissues (*P* < 0.05).

### 3.6 Color assessment of cooked shrimp

**Figure 4:**
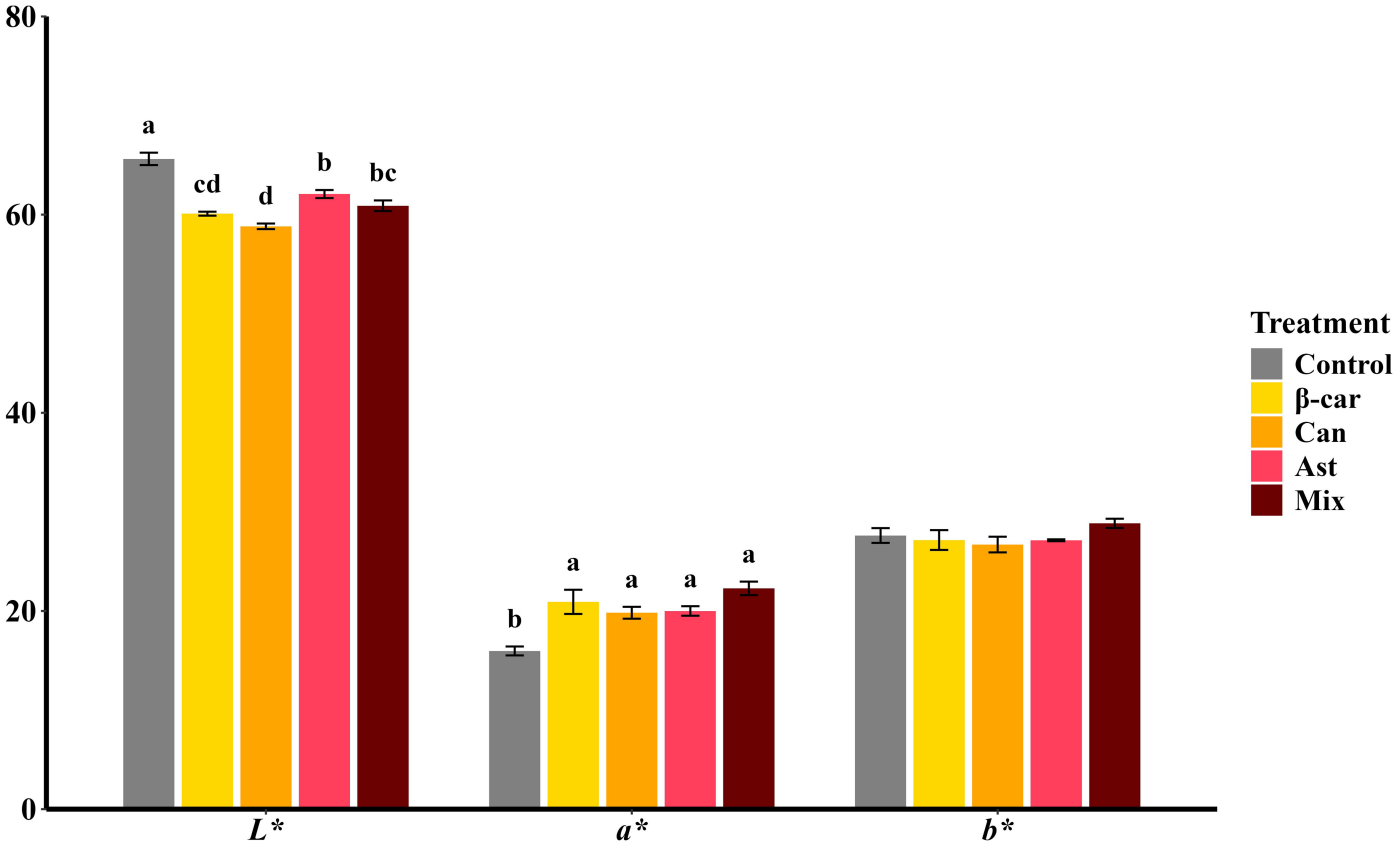
Color parameters of cooked whole-body shrimp fed diets containing different carotenoids. Values are expressed as means ± S.E.M. (n = 3). Bars with different letters represent significant differences among various treatments (*P* < 0.05). ANOVA *P* > F: L*(< 0.001), a* (0.002), b* (0.302).

Color parameters (CIELAB) of cooked *P. vannamei* after 56 days are shown in **Figure 4**. Diets containing β-carotene, astaxanthin, or canthaxanthin improved coloration relative to the control, evidenced by lower L* and higher a* values (both *P* < 0.05), while b* was unchanged (*P* > 0.05). The mixture diet showed the same pattern, and a* did not differ from the single-carotenoid diets (*P* > 0.05). Carotenoid profiling confirmed that the mixture increased total tissue carotenoids; however, *P. vannamei* deposited most carotenoids in the hepatopancreas rather than in muscle. This tissue-specific distribution likely limited visible color gains, explaining why the mixture did not outperform single-carotenoid diets for a*.

### 3.7 Flavor of shrimp muscle

**Figure 5:**
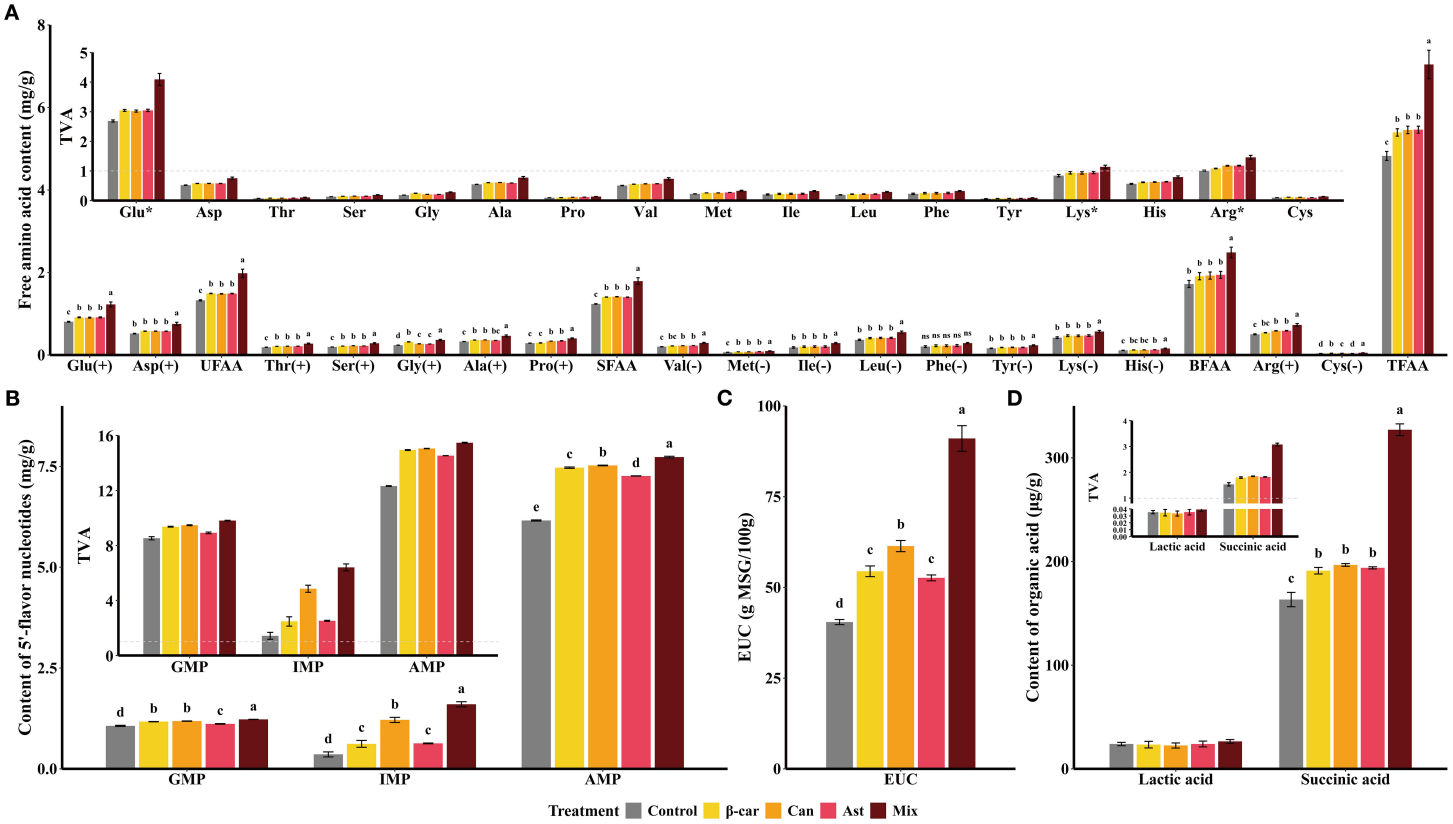
Changes of flavor profile in shrimp muscle induced by dietary carotenoids. Values are expressed as means ± S.E.M. (n = 3). Bars with different letters represent significant differences among various treatments (*P* < 0.05). ANOVA *P* > F: Glu (< 0.001), Asp (0.002), UFAA (0.302), Thr (< 0.001), Ser (< 0.001), Gly (< 0.001), Ala (< 0.001), Pro (< 0.001), SFAA (< 0.001), Val (< 0.001), Met (< 0.001), Ile (0.015), Leu (< 0.001), Phe (0.13), Tyr (< 0.001), Lys (0.009), His (< 0.001), BFAA (0.003), Arg (< 0.001), Cys (< 0.001), TFAA (< 0.001), GMP (< 0.001), IMP (< 0.001), AMP (< 0.001), EUC (< 0.001), Lactic acid (0.832), and Succinic acid (< 0.001). Free amino acids were categorized as follows: UFAA (umami free amino acids: Glu, Asp); SFAA (sweet free amino acids: Thr, Ser, Gly, Ala, Pro); BFAA (bitter free amino acids: Val, Met, Ile, Leu, Phe, Tyr, Lys, His); TFAA (total free amino acids). GMP, guanosine monophosphate; IMP, inosine monophosphate; AMP, adenosine monophosphate. (+), pleasant; (-), unpleasant; (*), TAV > 1.

The flavor profile of shrimp muscle after 56 days is shown in **Figure 5**. In **Figure 5A**, glutamate (Glu) and aspartate (Asp) were the most abundant free amino acids (FAAs); Glu had a taste‐activity value (TAV) > 1, indicating a major contribution to umami. Diets containing β-carotene, canthaxanthin, or astaxanthin significantly increased multiple FAAs (*P* < 0.05). However, the three carotenoids did not differ for umami FAAs (UFAA), sweet FAAs (SFAA), bitter FAAs (BFAA), or total FAAs (TFAA) (*P* > 0.05). The mixture diet further increased FAA contents (*P* < 0.05). As shown in **Figure 5B**, the umami-potentiating nucleotides inosine monophosphate (IMP), guanosine monophosphate (GMP), and adenosine monophosphate (AMP) all had TAVs > 1. All three nucleotides increased with carotenoid supplementation, with IMP higher in the canthaxanthin group than in the β-carotene and astaxanthin groups (*P* < 0.05); the mixture diet yielded the highest nucleotide levels overall (*P* < 0.05). The equivalent umami concentration (EUC; **Figure 5C**) rose with each carotenoid (*P* < 0.05), with canthaxanthin showing the largest gain (+51.79% vs. control). The mixture further increased EUC by 48.30% relative to canthaxanthin (*P* < 0.05). For organic acids (**Figure 5D**), succinic acid (umami) displayed TAV > 1 and increased with each carotenoid and with the mixture (*P* < 0.05), whereas lactic acid (sour) was quantified as a taste-related component. Overall, dietary carotenoids enhanced the umami of shrimp muscle, and the mixture provided additive benefits beyond single compounds.

### 3.8 Optimization of carotenoid blending ratios by RSM

Building on the optimal single-compound inclusion levels, the current study optimized carotenoid blend ratios using RSM. Dose ranges were β-carotene 200–500 mg/kg, canthaxanthin 100–300 mg/kg, and astaxanthin 50–250 mg/kg. RSM models were fitted with the following response variables: whole-body total astaxanthin (T-AST), whole-body total carotenoids (T-CAR), total antioxidant capacity (T-AOC), and CIELAB redness (a*).

**Figure 6:**
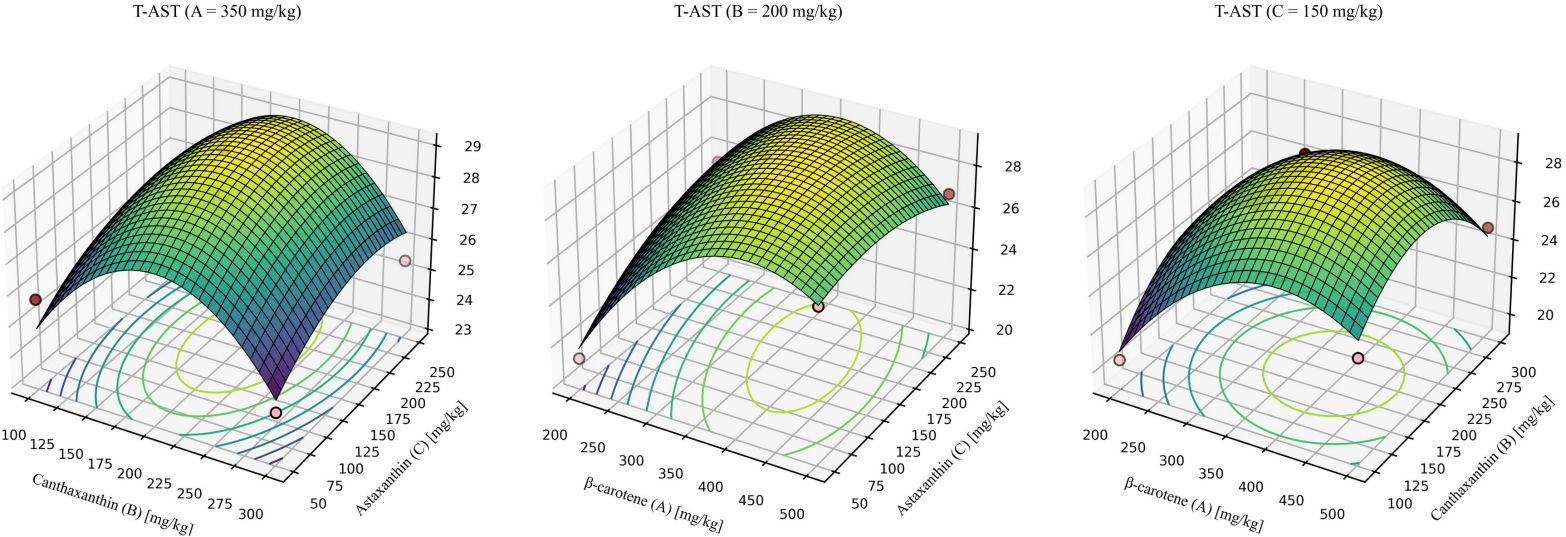
Response-surface model for whole-body total astaxanthin. Quadratic fit: T-AST = -12.84279 + 0.129226 (β-car) + 0.126550 (Can) + 0.057857 (Ast) - 0.000038 (β-car × Can) - 0.000074 (β-car × Ast) + 0.00000422244 (Can × Ast) - 0.000142 (β-car^2^) - 0.000283 (Can^2^) -0.000087 (Ast^2^). Where β-car, Can, and Ast denote the dietary concentrations (mg/kg) of β-carotene, canthaxanthin, and astaxanthin, respectively. Model statistics: overall *P* = 0.0135; R² = 0.941; lack-of-fit *P* = 0.342.

For T-AST (**Figure 6**), the model was significant with a non-significant lack of fit and high explanatory power (R^2^ > 0.9). The quadratic model was significant, showed no lack of fit, and explained most of the variance (R^2^ = 0.9412, Lack-of-fit *P* = 0.3418). The predicted optimum for maximizing T-AST was β-carotene: canthaxanthin: astaxanthin = 318.71:199.71:174.07 (mg/kg), yielding a maximum T-AST of 29.49 mg/kg.

**Figure 7:**
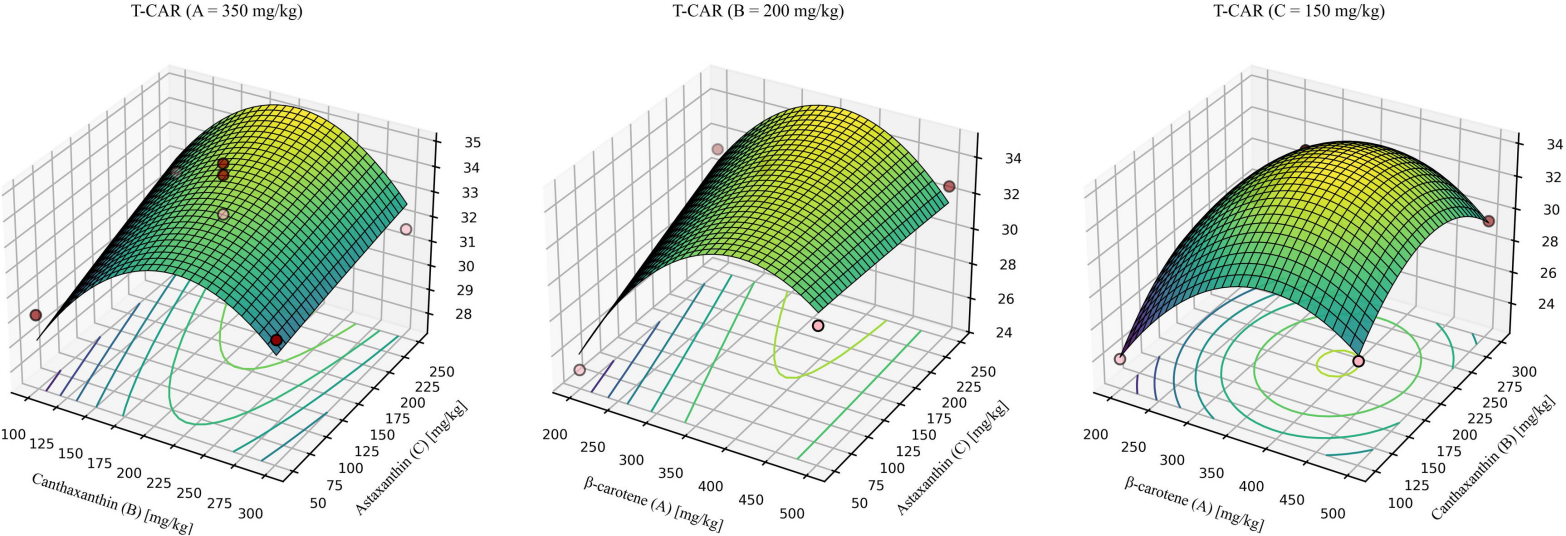
Response-surface model for whole-body total carotenoids. A = 350mg/kg, response surface at β-carotene fixed at 350 mg/kg. B = 200mg/kg, response surface at canthaxanthin fixed at 200 mg/kg. C = 150mg/kg, response surface at astaxanthin fixed at 150 mg/kg. Quadratic fit: T-CAR = -16.00748 + 0.162949 (β-car) + 0.144262 (Can) + 0.049764 (Ast) - 0.000030 (β-car × Can) - 0.000086 (β-car × Ast) - 0.000042 (Can × Ast) - 0.000188 (β-car^2^) - 0.000300 (Can^2^) - 0.00000475287 × (Ast^2^). Where β-car, Can, and Ast denote the dietary concentrations (mg/kg) of β-carotene, canthaxanthin, and astaxanthin, respectively. Model statistics: overall *P* = 0.0100; R² = 0.9482; lack-of-fit *P* = 0.3867.

For T-CAR (**Figure 7**), the model was significant with a non-significant lack of fit and high explanatory power (R^2^ > 0.9). The quadratic model was significant, showed no lack of fit, and explained most of the variance (R^2^ = 0.9482, Lack-of-fit *P* = 0.3867). The predicted optimum for maximizing T-CAR was β-carotene: canthaxanthin: astaxanthin = 361.73: 202.34: 236.23 (mg/kg), yielding a maximum T-CAR of 35.17 mmol/g.

**Figure 8:**
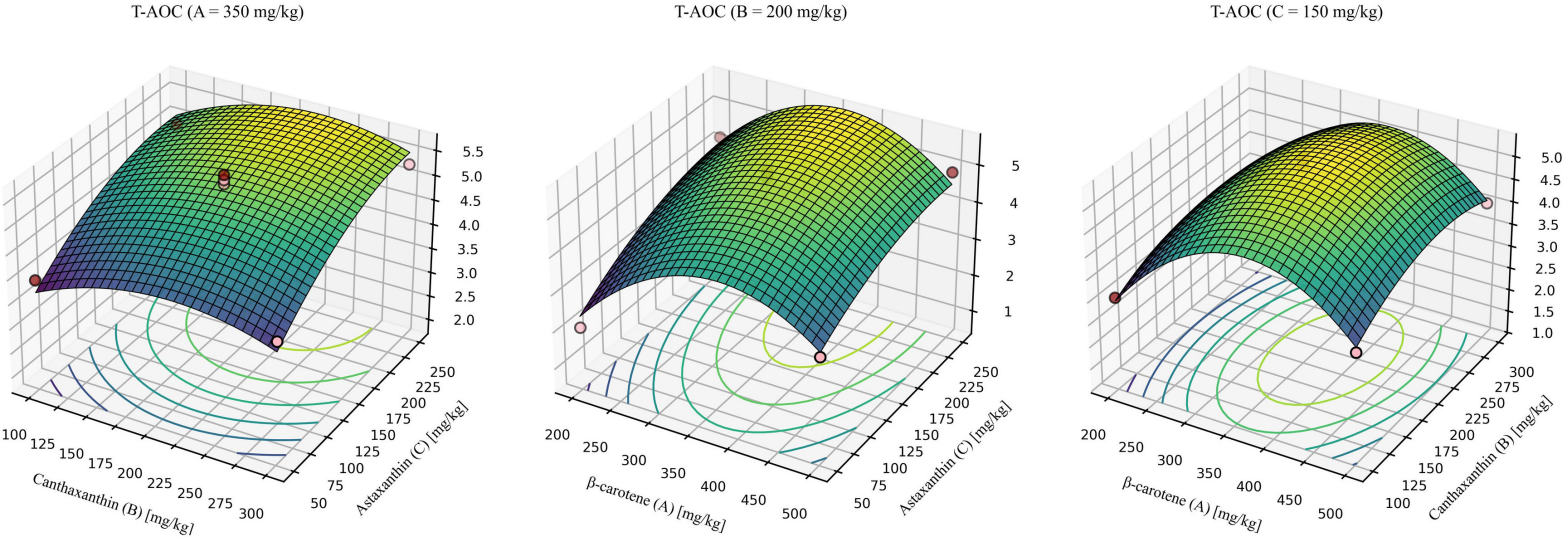
Response-surface model for total antioxidant capacity. A = 350mg/kg, response surface at β-carotene fixed at 350 mg/kg. B = 200mg/kg, response surface at canthaxanthin fixed at 200 mg/kg. C = 150mg/kg, response surface at astaxanthin fixed at 150 mg/kg. Quadratic fit: T-AOC = -7.76691 + 0.048888 (β-car) + 0.019508 (Can) + 0.020443 (Ast) + 0.00000574201 (β-car × Can) - 0.00000790276 (β-car × Ast) + 0.00000483255 (Can × Ast) - 0.000066 × (β-car^2^)- 0.000051 × (Can^2^) - 0.000038 × (Ast^2^) (*P* = 0.003, R^2^ = 0.9687, Lack-of-fit *P* = 0.0519).

For T-AOC (**Figure 8**), the model was significant with a non-significant lack of fit and high explanatory power (R^2^ > 0.9). The quadratic model was significant, showed no lack of fit, and explained most of the variance (R^2^ = 0.9687, Lack-of-fit *P* = 0.0519). The predicted optimum for maximizing T-AOC was β-carotene: canthaxanthin: astaxanthin = 354.17: 216.80: 192.12 (mg/kg), yielding a maximum T-AOC of 5.72 mmol/g.

**Figure 9:**
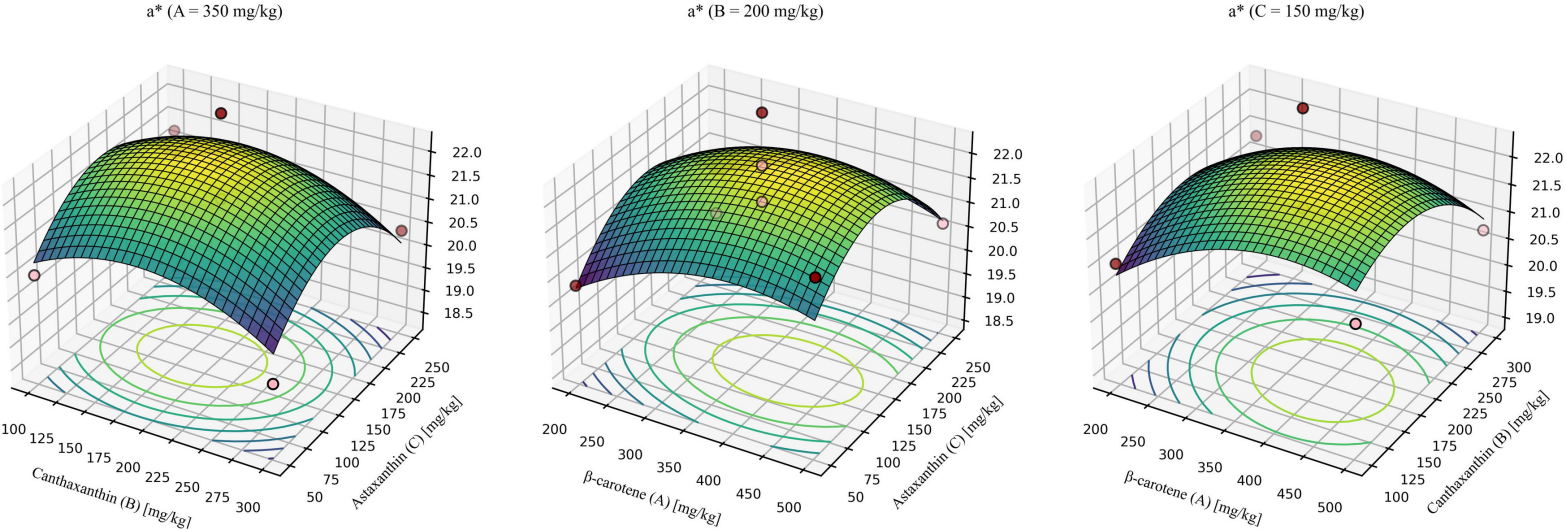
Response-surface model for redness (a*). A = 350mg/kg, response surface at β-carotene fixed at 350 mg/kg. B = 200mg/kg, response surface at canthaxanthin fixed at 200 mg/kg. C = 150mg/kg, response surface at astaxanthin fixed at 150 mg/kg. Quadratic fit: a* = 12.04752 + 0.023915 (β-car) + 0.032538 (Can) + 0.038081 (Ast) -0.000014 (β-car × Can) - 0.00000768056 (β-car × Ast) - 0.00000225 (Can × Ast) - 0.000026 × (β-car^2^) -0.000074 × (Can^2^) - 0.000120 × (Ast^2^) (*P* = 0.4945, R^2^ = 0.6570, Lack-of-fit *P* = 0.4650).

For redness (a*) (**Figure 9**), the model was not significant, indicating that within the tested inclusion ranges, carotenoid supplementation had a limited effect on a consistent with the single-factor comparisons.

**Figure 10:**
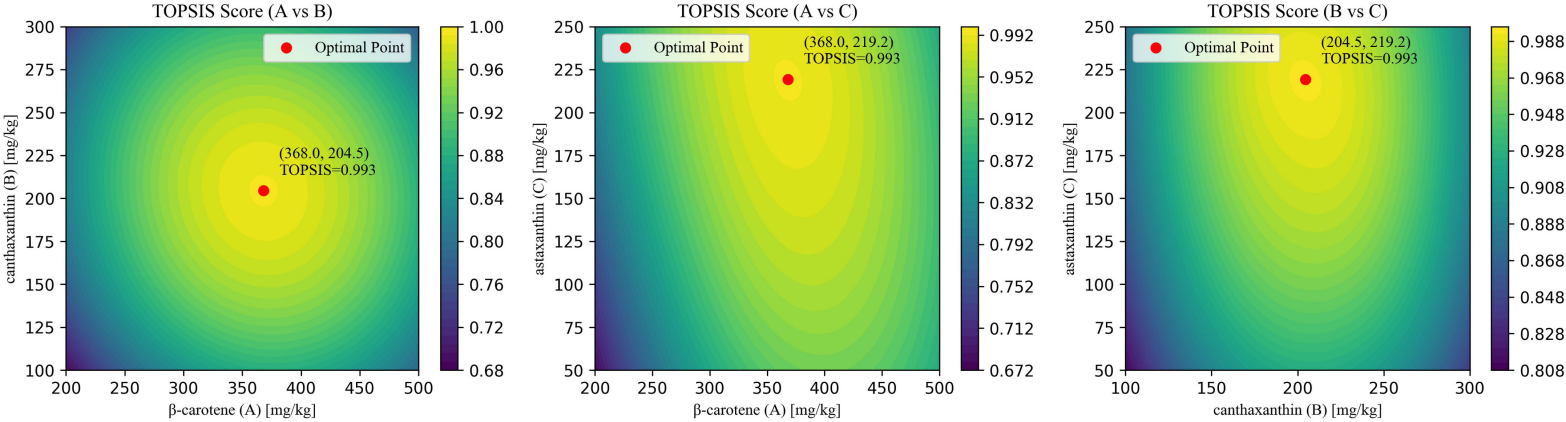
Results of AHP-CRITIC-TOPSIS scoring.

Using three significant responses-whole-body total carotenoids (T-CAR), whole-body total astaxanthin (T-AST), and total antioxidant capacity (T-AOC)-the current study assigned subjective weights with the analytic hierarchy process (AHP) and objective weights with the CRITIC method, then combined them by geometric averaging (**Supplementary Table S3**). The response-surface models were aggregated into a single composite score with TOPSIS. The optimal blend was β-carotene: canthaxanthin: astaxanthin = 368.0: 204.5: 219.2 mg/kg, yielding a TOPSIS score of 0.993 (**Figure 10**).

## 4 Discussion

Carotenoids are lipid-soluble pigments widely distributed in plants, animals, and microorganisms, with a characteristic C40 isoprenoid backbone (22, 23). In aquatic animals, particularly crustaceans, carotenoids are indispensable nutrients associated with body coloration, growth, reproduction, and embryonic development (2, 3, 24). However, these animals are unable to synthesize carotenoids *de novo*; instead, they must acquire them through dietary intake (25). Once ingested, carotenoids undergo limited metabolic transformations, including oxidation, reduction, isomerization, oxidative cleavage, and epoxide hydrolysis (26). β-Carotene, canthaxanthin, and astaxanthin share structural similarities and belong to the same metabolic pathway. Specifically, the β-ionone rings of β-carotene undergo ketolation at the 4,4′ positions to form canthaxanthin, which is subsequently hydroxylated at the 3,3′ positions to produce astaxanthin (27).

Our previous studies demonstrated that dietary supplementation with β-carotene (100–500 mg/kg) or canthaxanthin (50–400 mg/kg) increased the astaxanthin content in shrimp (6, 8). This indicates that although shrimp cannot synthesize β-carotene, they can convert β-carotene and canthaxanthin into astaxanthin. Moreover, supplementation with these precursors enhanced both antioxidant capacity and pigmentation. Accordingly, in this study we adopted supplementation levels reported as optimal in earlier studies to further evaluate both single and combined carotenoid supplementation (6, 8).

Analysis of carotenoid distribution in the muscle, hepatopancreas, and whole shrimp revealed significant tissue specificity, with the hepatopancreas serving as the primary storage site, consistent with previous findings (28). Regardless of the carotenoid supplemented, astaxanthin remained the predominant compound, further confirming the capacity of *P. vannamei* to bio-convert precursor carotenoids into astaxanthin. Notably, supplementation with β-carotene led to significantly higher astaxanthin accumulation in the hepatopancreas compared with direct astaxanthin supplementation, while combined supplementation exceeded the accumulation levels achieved by any single carotenoid. Unlike astaxanthin, β-carotene also functions as a vitamin A (VA) precursor, symmetrically cleaved by BCO1 to produce VA (29, 30). VA regulates fatty acid synthesis and, within a certain range, enhances lipid deposition (31). Because astaxanthin is largely stored in esterified form, the increase in VA from β-carotene supplementation may facilitate the synthesis of astaxanthin esters.

The unique poly-conjugated double-bond structure of carotenoids confers strong reactive oxygen species scavenging ability, making them key contributors to antioxidant defense (22). Malondialdehyde (MDA), a major end-product of lipid peroxidation, is commonly used to assess oxidative damage (32). Consistent with previous research, astaxanthin required a lower dietary supplementation level than β-carotene or canthaxanthin to achieve maximal antioxidant effects (6, 9, 33, 34). In the present study, astaxanthin at relatively low levels provided significantly higher total antioxidant capacity and lower MDA content compared with higher levels of β-carotene or canthaxanthin. This highlights the superior antioxidant efficiency of astaxanthin. Structural differences explain these effects: although β-carotene, canthaxanthin, and astaxanthin share a similar carbon skeleton, their terminal substituents determine singlet oxygen quenching capacity and antioxidant activity (35). The α-hydroxy-ketone groups at the termini of astaxanthin allow it to span lipid bilayers, stabilizing membranes and enhancing resistance to lipid peroxidation (36) Moreover, synergistic effects have been reported between carotenoids (37). Our results reaffirm this, showing that combined supplementation of β-carotene and astaxanthin enhanced antioxidant capacity more effectively than either alone (6). Importantly, the combined supplementation of all three carotenoids at their optimal levels did not produce negative outcomes associated with excessive carotenoid intake; instead, it exceeded the benefits of single supplementation.

The application of nutritional geometry has advanced understanding of nutrient interactions by modeling optimal dietary balances (38, 39). Jiang et al. (40) applied response surface methodology to analyze combined effects of astaxanthin and β-carotene on Chinese mitten crab (*Eriocheir sinensis*). Building on this, we employed response surface methodology to model shrimp responses in terms of carotenoid deposition, pigmentation, and antioxidant performance. Multi-criteria decision-making was then applied to integrate results. The TOPSIS method (Technique for Order Preference by Similarity to the Ideal Solution) determines optimal solutions based on proximity to ideal benchmarks (41, 42). To derive indicator weights, we combined AHP (a subjective weighting method using hierarchical structuring and pairwise comparison (21) with CRITIC (an objective weighting method based on indicator variability and conflict (20)). This approach balanced expert judgment with data-driven evidence. Using this framework, we identified an optimal dietary ratio of β-carotene: canthaxanthin: astaxanthin = 368.0: 204.5: 219.2 mg/kg.

Nevertheless, limitations remain. Our design did not constrain the total carotenoid inclusion level. Although combined supplementation enhanced antioxidant activity, pigmentation, and muscle flavor, it substantially increased additive costs. From a practical perspective, if low-dose combinations of carotenoids can outperform equivalent doses of a single carotenoid-especially astaxanthin-such strategies may improve both biological performance and economic feasibility.

## 5 Conclusion

In conclusion, this study demonstrates an effective strategy to improve growth performance and product quality in *P. vannamei*. In a 56-day feeding trial, β-carotene, canthaxanthin, and astaxanthin-each supplemented at literature-based levels-consistently enhanced growth performance, elevated digestive enzyme activities, and strengthened antioxidant status, with the combined treatment yielding additional benefits. Tissue profiling revealed that supplementation promoted esterification; β-carotene stimulated greater total carotenoid and astaxanthin deposition than direct astaxanthin, and the mixture produced the highest tissue concentrations. Color improved (lower L*, higher a*), although the modest and plateauing increase in a* was consistent with deposition primarily in the hepatopancreas rather than muscle. Flavor quality also improved, as reflected by increased levels of umami-related free amino acids and nucleotides, which elevated the equivalent umami concentration. Response-surface modeling integrated with AHP–CRITIC–TOPSIS identified an optimized blend (β-carotene: canthaxanthin: astaxanthin = 368: 204.5: 219.2 mg/kg; TOPSIS = 0.993) that maximized carotenoid deposition and antioxidant capacity within the tested ranges. Collectively, these results provide a rigorously benchmarked, optimization-guided foundation for designing carotenoid blends that deliver biological and sensory benefits in intensive shrimp culture. Nevertheless, a limitation of this study is that optimization did not impose a fixed total-carotenoid or cost constraint. Future research will incorporate cost-aware, multi-objective optimization under defined dose and cost caps to identify minimal-effective blends and validate them across production systems and seasons.

## Data Availability

The original contributions presented in the study are included in the article/**Supplementary Material**. Further inquiries can be directed to the corresponding author.
