# Supplementary material for: Comprehensive evaluation of individual and combined dietary carotenoids in Penaeus vannamei and response surface analysis for optimizing combinations
Source: Front Immunol. 2025 Oct 15;16:1688761. doi: 10.3389/fimmu.2025.1688761 (PMC12568400; doi:10.3389/fimmu.2025.1688761)
Supplement: Supplementary file 1 [file DataSheet1.docx]

**Table S1**. Response surface methodology design to refine dietary supplementation carotenoids.

| **Treatment** | **Factors (mg/kg)** | | |
| --- | --- | --- | --- |
|  | **β-car** | **Ast** | **Can** |
| 1 | 350 | 150 | 200 |
| 2 | 350 | 150 | 200 |
| 3 | 350 | 50 | 100 |
| 4 | 200 | 50 | 200 |
| 5 | 500 | 150 | 300 |
| 6 | 500 | 250 | 200 |
| 7 | 200 | 150 | 100 |
| 8 | 350 | 150 | 200 |
| 9 | 200 | 150 | 300 |
| 10 | 350 | 150 | 200 |
| 11 | 200 | 250 | 200 |
| 12 | 350 | 150 | 200 |
| 13 | 500 | 50 | 200 |
| 14 | 350 | 250 | 100 |
| 15 | 500 | 150 | 100 |
| 16 | 350 | 50 | 300 |
| 17 | 350 | 250 | 300 |

**Table S2**. Effect of different dietary carotenoids on carotenoid compositions in *P. vannamei.*

| **Parameters^1^** | **Treatments** | | | | | ***P*-value** |
| --- | --- | --- | --- | --- | --- | --- |
|  | **Control** | **β-car** | **Can** | **Ast** | **Mix** |  |
| **Whole shrimp** |  |  |  |  |  |  |
| Free astaxanthin | 3.81^d^±0.07 | 6.38^b^±0.04 | 4.92^c^±0.18 | 4.94^c^±0.03 | 7.57^a^±0.04 | < 0.001 |
| Ester-astaxanthin | 0.31^e^±0.05 | 14.46^b^±0.61 | 6.27^d^±0.05 | 8.08^c^±0.31 | 21.67^a^±0.45 | < 0.001 |
| Total astaxanthin | 4.12^e^±0.09 | 20.84^b^±0.65 | 11.19^d^±0.16 | 13.02^c^±0.34 | 29.24^a^±0.49 | < 0.001 |
| Zeaxanthin | 0.23^d^±0.03 | 1.01^b^±0.06 | 0.55^c^±0.08 | ND | 1.47^a^±0.12 | < 0.001 |
| Canthaxanthin | ND | 0.22±0.02 | ND | ND | ND | - |
| Echinenone | ND | 0.20±0.05 | ND | ND | ND | - |
| β-carotene | ND | 1.41±0.16 | ND | ND | 1.39±0.03 | 0.925 |
| Total carotenoid | 4.35^d^±0.06 | 24.5^b^±0.42 | 11.97^c^±0.23 | 13.02^c^±0.34 | 34.36^a^±0.6 | < 0.001 |
| **Hepatopancreatic** |  |  |  |  |  |  |
| Free astaxanthin | 2.80^e^±0.27 | 30.34^b^±0.17 | 15.31^c^±0.16 | 14.12^d^±0.11 | 32.31^a^±0.02 | < 0.001 |
| Ester-astaxanthin | 0.35^e^±0.17 | 159.83^b^±1.19 | 121.52^c^±1.25 | 83.32^d^±0.16 | 315.98^a^±3.71 | < 0.001 |
| Total astaxanthin | 3.15^e^±0.16 | 190.17^b^±1.12 | 136.82^c^±1.09 | 97.44^d^±0.05 | 348.29^a^±3.72 | < 0.001 |
| Zeaxanthin | ND | 8.78^b^±0.27 | 2.28^c^±0.07 | ND | 30.7^a^±2.21 | < 0.001 |
| Canthaxanthin | ND | 1.35^b^±0.12 | ND | ND | 2.04^a^±0.13 | < 0.001 |
| Echinenone | ND | 1.84^b^±0.05 | 0.92^c^±0.09 | 0.68^d^±0.07 | 9.20^a^±0.07 | < 0.001 |
| β-carotene | 0.48^b^±0.14 | 11.5^a^±0.27 | 0.90^b^±0.12 | 1.03^b^±0.2 | 11.30^a^±0.01 | < 0.001 |
| Total carotenoid | 3.63^e^±0.19 | 241.97^b^±2.04 | 158.74^c^±1.09 | 109.89^d^±0.35 | 500.91^a^±8.39 | < 0.001 |
| **Muscle** |  |  |  |  |  |  |
| Free astaxanthin | 0.67^d^±0.03 | 1.21^b^±0.01 | 1.10^c^±0.01 | 1.12^c^±0.01 | 2.42^a^±0.01 | < 0.001 |
| Ester-astaxanthin | 0.17^c^±0.02 | 0.58^b^±0.16 | 0.61^b^±0.16 | 0.86^b^±0.16 | 1.52^a^±0.03 | < 0.001 |
| Total astaxanthin | 0.84^c^±0.05 | 1.78^b^±0.15 | 1.71^b^±0.16 | 1.99^b^±0.16 | 3.94^a^±0.03 | < 0.001 |
| Zeaxanthin | ND | 0.08^b^±0.01 | 0.07^b^±0.01 | ND | 0.19^a^±0.01 | < 0.001 |
| β-carotene | ND | 0.22^b^±0.02 | ND | ND | 0.32^a^±0.01 | < 0.001 |
| Total carotenoid | 0.77^c^±0.01 | 2.25^b^±0.14 | 1.99^b^±0.18 | 2.22^b^±0.16 | 5.18^a^±0.02 | < 0.001 |

^1^ Values are expressed as means ± S.E.M. (n = 3). Different superscript letters within the same row indicate statistically significant different (*P* < 0.05). ND: not detected.

**Tables S3**. Weight of response variables derived by AHP and CRITIC and the combined weight used in TOPSIS.

| **Parameters**^1^ | **Weight of response variables** | | |
| --- | --- | --- | --- |
|  | **Subjective** | **Objective** | **Comprehensive** |
| T-AST | 0.4053891 | 0.2380712 | 0.318423 |
| T-CAR | 0.113969 | 0.2491015 | 0.1727017 |
| T-AOC | 0.4806419 | 0.5128273 | 0.5088753 |

^1^T-AST, whole-body total astaxanthin; T-CAR, whole-body total carotenoids; T-AOC, total antioxidant capacity.
